# The role of community health workers in improving HIV treatment outcomes in children: lessons learned from the ZENITH trial in Zimbabwe

**DOI:** 10.1093/heapol/czx187

**Published:** 2018-01-03

**Authors:** Joanna Busza, Ethel Dauya, Tsitsi Bandason, Victoria Simms, Chido Dziva Chikwari, Memory Makamba, Grace Mchugh, Shungu Munyati, Prosper Chonzi, Rashida A Ferrand

**Affiliations:** 1 Department of Population Health, London School of Hygiene & Tropical Medicine, London WC1E 7HT, UK; 2 Biomedical Research and Training Institute, Harare, Zimbabwe; 3 Independent Consultant, Zimbabwe; 4 City of Harare Health Services, Harare, Zimbabwe

**Keywords:** Community health workers, antiretroviral treatment, adherence, children, caregivers, HIV, social support, Zimbabwe

## Abstract

Reliance on community health workers (CHWs) for HIV care continues to increase, particularly in resource-limited settings. CHWs can improve HIV service use and adherence to treatment, but effectiveness of these programmes relies on providing an enabling work environment for CHWs, including reasonable workload, supportive supervision and adequate training and supplies. Although criteria for effective CHW programmes have been identified, these have rarely been prospectively applied to design and evaluation of new interventions. For the Zimbabwe study for Enhancing Testing and Improving Treatment of HIV in Children (ZENITH) randomized controlled trial, we based our intervention on an existing evidence-based framework for successful CHW programmes. To assess CHWs’ experiences delivering the intervention, we conducted longitudinal, qualitative semi-structured interviews with all 19 CHWs at three times during implementation. The study aimed to explore CHWs’ perceptions of how the intervention’s structure and management affected their performance, and consider implications for the programme’s future scale-up and adoption in other settings. CHWs expressed strong motivation, commitment and job satisfaction. They considered the intervention acceptable and feasible to deliver, and levels of satisfaction rose over interview rounds. Intensive supervision and mentoring emerged as critical to ensuring CHWs’ long-term satisfaction. Provision of job aids, standardized manuals and refresher training were also important, as were formalized links between clinics and CHWs. Concerns raised by CHWs included poor remuneration, their reluctance to stop providing support to individual families following the requisite number of home visits, and disappointment at the lack of programme sustainability following completion of the trial. Furthermore, intensive supervision and integration with clinical services may be difficult to replicate outside a trial setting. This study shows that existing criteria for designing successful CHW programmes are useful for maximizing effectiveness, but challenges remain for ensuring long-term sustainability of ‘task shifting’ strategies.

Key Messages

- Use of CHWs to deliver HIV care and support programmes is increasing.
- Existing literature highlights key criteria for effectiveness of CHW-delivered programmes.
- As part of a randomized controlled trial in Zimbabwe, we designed and evaluated a CHW intervention providing adherence support to families of children living with HIV based on identified best practices.
- Recruiting experienced CHW, providing intensive training, supervision and mentoring, and linking them to clinic staff resulting in high levels of job satisfaction and motivation.
- Challenges related to CHW perceptions of inadequate remuneration, concerns about time-limited support to households and lack of longer-term sustainability.

## Introduction

Reliance on community health workers (CHWs) to support care for long-term conditions such as HIV continues to increase, particularly in resource-limited settings (Celletti *et al.* 2010; Lewin *et al.* 2010). The benefits of engaging this cadre of health providers include their familiarity with local issues, rapport with community members, and lower human resource costs (Torpey *et al.* 2008). The latter is often a justification for ‘task shifting’, where roles that previously were seen to require clinical skills have been transferred to lay personnel who are often volunteers, freeing up more expensive and higher level staff (Zachariah *et al.* 2009). Furthermore, CHWs provide critical support where human resources remain scarce (Rasschaert *et al.* 2011; Masquillier *et al.* 2016).

CHWs perform multiple functions in HIV programmes, including referring community members for HIV testing, linking them to care, accompanying them to clinic appointments, providing psychosocial support and making referrals to other services (Gusdal *et al.* 2011; Thomson *et al.* 2013). CHWs have been shown to improve uptake of HIV services and treatment adherence in diverse settings (Franke *et al.* 2012; Wouters *et al.* 2012). CHW programme effectiveness depends on an enabling work environment for CHWs, including workload, supportive supervision, supplies and equipment and respect from community members (Jaskiewicz and Tulenko 2012), as well as locally supportive social norms and health policies (Kok *et al.* 2015).

A review by Hermann *et al* (2009) drew on the extensive literature on CHWs to identify 10 criteria for effective CHW programmes, summarized in Table 1 (Hermann *et al.* 2009). Among these, five were considered necessary for successful implementation, three for improving quality, while two are specific to supporting provision of antiretroviral therapy (ART) in the context of HIV.

**Table 1.** Criteria for successful CHW programmes (Hermann *et al* 2009)

Basic essential conditions	Description
Selection and motivation	CHW need to understand the community in which they work and be trusted Formal education less important than motivation to work in community
Initial training	Content and length should be based on existing knowledge and experience Should be participatory and focus on practical skills and problem-solving Communication and counselling should be emphasized
Simple guidelines and standardized protocols	Materials should ensure CHW cover all areas in which they have been trained Manuals prevent CHW feeling overwhelmed by multiple tasks Can be used as basis for supportive supervision
Supervision and support	Regular mentoring and refresher training maintain motivation Structured and constructive supervision maintains programme quality
Adequate remuneration/career structure	Remuneration in some form crucial for CHW to feel valued Compensation increases CHW commitment and reduces drop-out
**Required for scale-up**	
Political support	Formal role of CHW needs to be defined over time and regulated within broader health system
Alignment with health system strengthening	Functioning health system required for CHW to function effectively CHW should make referrals to comprehensive constellation of services
Flexibility and dynamism	Programmes should evolve and adapt as social and health conditions change
**Specific to ART support**	
Using experience of people living with HIV	CHW living with HIV offer hope and inspiration and can lead by example
Focus on chronic care, retention and adherence	Self-management and sustainability of care key issues to address by CHW CHW should emphasize skills for long-term retention in care and adherence

These criteria reflect lessons distilled from existing programmes, but to date have not been prospectively applied in the design and evaluation of newly introduced CHW interventions. As part of the Zimbabwe study for Enhancing Testing and Improving Treatment of HIV in Children (ZENITH) randomized controlled trial, we applied these criteria during the development of the CHW-delivered intervention in an effort to adhere to identified best practices and maximize chances of programme effectiveness.

The ZENITH trial tested a community-based intervention offering CHW home visits to caregivers of children living with HIV in seven high-density communities in Harare, Zimbabwe. The intervention was based on evidence suggesting that home visits may be an effective mechanism for providing social support and counselling to improve retention in HIV care and adherence to ART among adults and children (Van Winghem *et al.* 2008; Etienne *et al.* 2010; Muñoz *et al.* 2010; Bain-Brickley *et al.* 2011; Alamo *et al.* 2012; Grimwood *et al.* 2012). Results of the trial showed significantly reduced virological failure among children aged 6–15 years who received CHW home visits (Ferrand *et al.* 2017).

The development and content of the ZENITH intervention are described elsewhere (Busza *et al.* 2014). In summary, CHWs provided 12–15 structured household visits adapted from an existing strengths-based case management approach (CDC 2011) to support caregivers at key points in children’s HIV care such as diagnosis and ART eligibility assessment, treatment initiation, disclosure of HIV status to the child and others, and management of long-term adherence. CHWs also liaised with specialist study nurses deployed at each of the primary care clinics to link community and facility care. Nurses would contact CHWs when they identified potential problems, such as when enrolled children missed appointments, while CHWs followed up with nurses if they felt children were at risk of treatment non-compliance.

As part of a process evaluation of the ZENITH trial, we reviewed the intervention’s performance against the Hermann *et al* criteria, with specific attention to CHWs’ own perceptions and experiences. We present findings from a qualitative study of how CHWs perceived their work, and how they felt their job satisfaction and performance were affected by different aspects of the programme’s structure and management. The aim was to give a voice to the central actors of a CHW programme that successfully met its goals, reflect on how use of a criteria-based framework during intervention design affected CHWs’ delivery, job satisfaction and motivation, and consider implications for the programme’s future scale-up and adoption in other settings.

## Materials and methods

We conducted longitudinal semi-structured qualitative interviews with the 19 CHWs who delivered the ZENITH intervention at three time periods during the trial: baseline (following recruitment and training but before home visits work started), midline (after 1 year of implementation of the intervention) and at the end of the intervention 2 years later, prior to unblinding.

The interview guides explored CHWs’ positive and negative experiences, and their views on the intervention’s structure, materials and procedures, and training. At baseline, questions also asked about the decision to apply to be a CHW, expectations of the work and early concerns, while at the final interview, CHWs were asked to reflect on the intervention as a whole, provide their own views of its effectiveness, and comment on their participation.

An independent social scientist, not involved in trial design or implementation, interviewed CHWs in their homes or one of the study clinics. Interviews were conducted in Shona and lasted approximately 1 h. After obtaining written informed consent, the researcher recorded interviews and later transcribed them verbatim, followed by translation into English.

During thematic content analysis we used NVIVO 10 to code transcripts into *a priori* and data-driven themes, comparing these over the three interview rounds to identify changes in CHWs’ perceptions across the intervention period. For this study, further coding was conducted along the 10 criteria proposed by Hermann *et al.*

Ethical approval was obtained from the London School of Hygiene & Tropical Medicine, the Medical Research Council of Zimbabwe and the Biomedical Research and Training Institute, Harare. To avoid inadvertently identifying individuals, we provide interview number and sex for participant quotes.

## Results

Out of 20 CHWs initially engaged, 1 dropped out early in the programme for personal reasons and we completed all 3 interviews with the remaining 19. CHWs were each responsible for an average of nine children (range 5–15 years). Among the 19 CHWs, 17 were female. We present our findings by each of the Herman *et al.* criteria to facilitate assessment of whether our attempts to follow identified best practices achieved high levels of CHW motivation, satisfaction and perceptions of programme effectiveness.

### Selection and motivation

The trial intervention was delivered by a local organization, the Child Protection Society (CPS), which already employed a pool of CHWs trained in HIV counselling and home-based care. CPS had worked in three of the study communities since 2000. CPS was subcontracted to identify eligible volunteers from within their existing pool, ensuring the intervention was delivered by CHWs from the locality, who had relevant experience and a track record of sustained motivation for community work.

Interviews at baseline asked about CHWs’ decision to work for ZENITH and their expectations. CHWs felt well-prepared for ZENITH, which they saw as similar to other CPS programmes.

As volunteers who were doing work with CPS, when we were introduced to ZENITH we just said, ‘well we have always been doing this’. Therefore, there is no problem to continue working with young people. (Baseline, Male #2)

A recurring explanation for CHW interest in the programme was empathy and ‘passion’ for helping others, particularly children. This passion was enhanced among those who described living with HIV themselves or having cared for relatives and thus wanting to share their expertise or ‘give something back’, especially if they felt they had benefitted from community support.

What pushed us is passion for the children that we have. … Passion! [and] the background from where we came. We were less privileged children and we have people who helped us. As I was growing up I would say God, I would like to help other people for the sake of those that have helped me. (Baseline, Female #6)

I feel that I am doing something good so that I can help others … so that they are well and will live just like I have done. Because if I had not had someone who helped me I wouldn’t be where I am today. I would have died long back … I got support from other people [and] I feel I have to return that support to others. (Baseline, Female #1)

CHWs viewed ZENITH as an opportunity to develop their skills and stay ‘up to date’ with HIV programming. Building their own capacity was intertwined with CHWs’ pride in their work and confidence in their potential to contribute to society. They also appreciated that HIV care was a constantly evolving field, and new information would be required both to enhance their performance.

Since I have always been working with young people, I felt that if I were to join another programme I would get additional knowledge . … I would learn additional things that are important (Baseline Female #3)

Whenever I hear anything about HIV, I listen carefully so that I understand how it is progressing. Even if I see a paper that has been written about HIV, I really want to understand what is going on . … I wanted to gain enough knowledge about what I could do to help these children. (Baseline, Female #8)

We always need to be capacitated with knowledge when we are dealing with HIV/AIDS issues because they keep on changing. (Baseline, Male #2)

CHWs were also aware that programme funding tended to be short term and that keeping up with developments in prevention and treatment might help obtain future employment.

### Initial training, simple guidelines and standardized protocols

Given CHWs’ motivation to develop their skills, the programme’s training provided a key reason for volunteering. Two weeks of full-time training covered counselling skills, content of the home visit sessions, and issues specific to HIV among children and adolescents. After the first year of implementation a 1-week refresher course was provided which 17 CHWs (85%) attended.

CHWs enjoyed the training and asked for refresher sessions, stating they found the training to be more in-depth than others they had attended. The module on one-to-one counselling proved particularly popular, as did participatory ‘role plays’ to practice managing different types of situations.

The training was good because some of the things that we learnt were more advanced. We had never learnt about those things . … we would like to have regular refresher courses … because you forget some of the things. Therefore there is need to have regular refresher courses and to continue learning. (Baseline, Female #7)

We also learnt about counselling. You should let a person speak while you are listening. Don’t tell them what to do. Listen very carefully and repeat the words they would have spoken, as a way of showing you have understood. (Baseline, Female #4)

They would ask us to watch when people were acting. Suppose I am visiting a child - how do I approach the house? How do I start talking to the people that are at this house? … Therefore we learnt a lot even about approaching houses. Those dramas were really helpful. (Baseline, Female #3)

Yet there was also some frustration over the length of the training as it interfered with income-earning and household activities, with no compensation.

If the trainings become too long our business will go under, yet we are not getting anything from ZENITH. (Baseline, Female #9)

Two weeks was just too long, I would have wanted it to be a week. (Baseline, Male #2)

Each CHW received a manual detailing the objectives and procedures for each visit and case management forms to complete for each session. The manual reminded CHWs of the order, timing and activities for household visits and was available in English and Shona. CHWs felt such standardization offered a sense of security in that they did not have to improvise but could follow a clear visit structure at each stage of supporting a household.

If I realise that I am getting lost, I will open my manual and read. Eh, I will open my manual and this will bring me back. I would then know that ‘oh, I am supposed to do this and that’. Eh, ah, the manual really helps me (Midline, Female #2)

Additional job aids included a picture book for children about HIV and ART and pill-count charts. These were well-received although CHWs wanted more child-friendly materials and gifts to leave with households, partly to help gain access and build rapport, and partly because CHWs were cognizant of the pervasive poverty among the households they visited.

We should get something that entertains the children so that when you talk to them, they will then do something when you are gone. A toy or something … when they see the toys, they will know that this person taught them about this and that, and she gave me a toy. (Midline, Female #7)

Interestingly, at the start of the trial, several CHWs asked for ‘branded’ goods to identify them as part of the team. Suggestions included T-shirts, hats or ID cards, which CHWs believed would prevent them being viewed with suspicion by households, or even accused of being political agents.

When I arrive and people see my I.D. or if I have a t-shirt or a hat … they will know that this person wants to talk about health issues. (Baseline, Female #2)

[If] they can see that I am wearing a badge which has got my picture and is also saying ZENITH, they will understand. These days we are facing a challenge of politics . … And when they see me knocking it would appear as if I am campaigning. (Baseline, Female #1)

By the end of the programme, however, CHWs had changed their minds, realizing that caregivers did not appreciate identifying clothing because it alerted others to the household having an HIV-positive member, due to ZENITH’s focus on HIV. In fact, looking anonymous proved to be an advantage as CHWs could pretend to be a friend or relative when confronted by neighbours or other family members, particularly when they knew the caregiver was worried about being stigmatized for having HIV in the family.

We had one ZENITH t-shirt made for us. So someone might tell you ‘when you are coming to my house, you should come wearing things that do not look like a uniform’. (Final interview, Female #11)

She said ‘when you are coming to me, I do not want you to put on t-shirts showing HIV things’. So this was something that she wanted in order to protect herself so that it is not known that she has a child who is living with HIV. (Final interview, Female #19)

### Remuneration, career structure and supervision

CHWs raised the issue of compensation at all three rounds of data collection. Although ZENITH provided a monthly stipend of $20, CHWs were considered volunteers. CHWs accepted this, describing being motivated by other factors, but nonetheless would have preferred better financial recognition of their work. Most requested an increase in the stipend, although others wanted proof of their work credentials to use in securing future employment. Occasional gifts, such as rain jackets and Christmas hampers, proved popular tokens and helped mitigate financial requests.

We were expecting to be given better remuneration … it turned out to be too little but it did not affect the work . …We were thinking that probably it would go up to $40 … [but] it went up to $25 (Final interview, Female #3)

I need the [training] certificate because like I mentioned before, I did not go to school. So getting a certificate really encourages me. (Baseline, Female #2)

I want to appreciate ZENITH. Last time they bought us packs for Christmas. This is something that I enjoyed and it lifted us up. We felt that they appreciated us . … . We thought they appreciated us a lot so we are just saying ‘thumbs up!’ (Midline, Female #14)

Intensive supervision to ensure quality was a key component of the intervention. The CHW supervisor held monthly meetings to check on progress, receive updates on visited households, share challenges and solutions within the team, and hear grievances such as dissatisfaction with pay. At first, CHWs found these meetings long and boring, but over time appreciated their role in bringing colleagues together to discuss and improve work practices.

If there were any challenges, we would do those meetings at the end of the month. Each person will be sharing her views … ‘why don’t you do this and that?’ So it was helpful to us … And indeed they [CHWs] would do what we discussed and things would move forward (Final interview, Female #6)

The supervisor herself was well-liked; CHWs particularly praised the extra efforts she made in helping with difficult cases, e.g. accompanying CHWs on household visits or following up by phone.

When I didn’t know something, I would ask … when we went to give our reports to [CHW supervisor]. She would say … ‘you are supposed to do this, you do this and that’. It was simple. I now knew what I was supposed to be doing . … What [she] would do is if she has noticed that a child had a need, she would also visit caregivers, she visited with me. (Final interview, Female #1)

### Issues around sustainability: political will, health systems and flexibility

CHWs expressed concern over limitations of the ZENITH trial due to it being ‘research’. CHWs felt they were reaching only a small proportion of families in need, and for insufficient time. CHWs expressed discomfort about randomization to a control group, whereby children known to be living with HIV did not receive CHW support.

As a result, the importance of sustainability and scale-up were salient to CHWs from the start, and they repeatedly shared their hopes that the programme’s future would be secured through new funds. Others tried to think of creative ways to encourage participants to ‘cascade’ their knowledge through the community to reach those who were unable to enrol.

I feel … only a few kids are being enrolled in the program . … In my opinion this program is good. If only money could be found, we would enrol every child in the programme - every child who has tested positive to HIV. Then we would do the follow-up that we are doing for these other children who were selected. (Midline, Female #9)

If the people with whom we worked manage to share the information that we left them throughout the community with the others who did not get the opportunity to be enrolled … it would help other people in the community, yes. (Final interview, Male #2)

The end of the trial resulted in further anxiety among CHWs, who sometimes felt they were abandoning families with whom they had developed close relationships. Although the home visit programme was designed to last 18 months, with final visits devoted to working with caregivers on sustaining long-term management of children’s care, CHWs were nonetheless worried that children’s health would suffer. They believed households had become dependent on their assistance and might struggle to maintain the same level of engagement following completion. Some families were considered more ‘successful’ and CHWs were confident that these had become self-reliant. It particularly pained them, therefore, that families most in need and struggling to benefit from the intervention would be those to experience negative consequences of the end of home visits.

This is going to affect so many children. They will become sick because they do not have anyone to encourage them. (Final interview, Female #4)

I was thinking that it could have gone on for 5 years so that people will get used to it. We only did it for a very short time (Final interview, Female #6)

Among families that accepted this programme, I feel that that they are not going to have challenges with regards to taking medication. (Final interview, Female #12)

Because CHWs were familiar with individual household circumstances, they differentiated between families that could safely ‘graduate’ and those that required further attention, and were thus frustrated by the lack of longer-term commitment to the intervention.

### The role of people living with HIV

While living with HIV was not a requirement for recruitment, six CHWs disclosed that they were living with HIV during baseline interviews, linking their HIV status to their motivation for joining the programme.

I am one of those people. I am on medication, so I am encouraging children to continue taking their medication on time. (Baseline, Female #12)

CHWs described how they used their personal experiences to benefit other households, particularly when they had overcome similar challenges to those facing caregivers. For some, it felt therapeutic to help others.

I was a troubled person when I got tested. There was a time when I was really distressed. I met this person whom I talked to. … This person told me that they were found with HIV and were cared for by other people. I realized that this was good and I decided to copy their attitude . … When I see a sick person who has HIV I will talk to them just like I was told and that is good. (Baseline, Female #8)

CHWs did not comment on whether they felt that living with HIV themselves made them better qualified for the role, however. We also did not systematically document to what extent CHWs disclosed their status to the caregivers they were supporting, and whether this was perceived to influence how the families responded to their advice.

### Emphasis on retention and adherence

One of the intervention’s primary goals was to increase children’s retention in care and adherence to treatment. Home visits centred around working with caregivers to overcome barriers to attending clinical appointments and collecting medication, establish ‘reminders’ for routine drug-taking, and build an enabling environment for the child’s care through appropriate referrals.

CHWs took a practical approach to household problems, and broadened their discussion to include a wide range of issues that they felt were preoccupying caregivers. They considered family matters to be determinants of caregivers’ successful management of children’s health, even if these were unrelated to HIV. For example, one CHW counselled a caregiver who was having relationship difficulties. Others discussed family conflicts and income-generating strategies with caregivers they visited.

You will then see a different issue from what you had gone there for . … It’s a personal problem that is in the family or it’s a family problem that is in the household. I will have to assist. I still have to counsel them because this will affect their taking of medication. (Midline, Female #12)

CHWs also forged close relationships with study nurses and would regularly discuss difficult cases with them to agree a coordinated response. They felt this regular exchange of information between nurses and CHWs provided a vital link between clinical and psychosocial care and strengthened programme effectiveness.

If children default or are refusing to go to the clinic, they [study nurses] always phone to say ‘can you come and assist us and explain this to the child? Like the case I am referring to, we had to go to talk to this girl … to say … ‘first of all, take your treatment seriously. You will have a healthy lifestyle just like anyone else but if you default on taking tablets as prescribed then you will have problems’ . … She is due for her visit today at the clinic. I will phone [the nurse] to find out whether she has come (Midline, Male #2)

## Discussion

According to CHWs, ZENITH fulfilled most criteria identified by Hermann *et al.* for well-functioning, CHW-delivered ART support. CHWs reported a positive experience and exhibited strong motivation, enduring commitment, and job satisfaction throughout the intervention. They considered the intervention both acceptable and feasible to deliver, and levels of satisfaction rose over the interview rounds as CHWs became more familiar with procedures and materials and attended refresher training. Over time, CHWs found some of their early concerns were unfounded or could be managed. For example, CHWs initially wanted uniforms or other items identifying them as health workers to avoid suspicions regarding their presence in communities. They soon learned, however, that this caused anxiety among caregivers, who feared being associated with HIV. Other CHW programmes have found this same attitude to uniforms during implementation (Masquillier *et al.* 2016). CHWs felt generally well received by households due to their familiarity with the local context and, for many, due to their personal experience of living with HIV or caring for others.

Feedback sessions ensured regular contact between CHWs and the study team, and provided opportunities for prompt management of emerging challenges. Intensive supervision, both in group settings and through individualized on-the-job mentoring, appeared to be a key component of ensuring long term satisfaction and commitment on the part of CHWs. Provision of job aids, standardized manuals, and refresher training were also deemed appropriate and useful. Finally, CHWs believed that their formalized links to clinics improved their ability to influence households. These aspects of the programme, however, were delivered in a trial setting where it was possible to provide intensive supervision and on-the-job mentoring, and ensure clinic nurses were in regular contact with CHWs about individual clients. This level of management is unlikely to be replicated at larger scale. Nonetheless, the importance CHWs attributed to support from their supervisor in this study mirrors similar findings from other research into determinants of good CHW performance and suggests that adequate supervision is essential to programme sustainability (Jaskiewicz & Tulenko 2012; Akintola and Chikoko 2016; De Neve *et al.* 2017).

The study used an existing pool of qualified, motivated and experienced CHWs. Although we recruited a relatively small number of CHWs, the high retention over the study period suggests that training and supervision met their needs. It is also possible, however, that the extremely high unemployment rate in Zimbabwe makes any formal work, even if mostly voluntary, attractive for its regular income and provision of training and skills that might be useful for future employment. In better economic times, fewer CHWs of similarly high calibre may be available, or they may require higher payment. Although literacy was not a prerequisite for selection of CHW, all were able to read and write in Shona. We relied on written materials for the training and provided all CHWs with a manual, including forms for each home visit that they had to fill out. CHWs found the manual helpful in reminding them of the order and details of expected activities. While it may not be practicable for CHW programmes to insist on literacy, we would recommend standardizing procedures through a manual, which increases the professionalization and accountability of CHWs.

A number of concerns were identified, particularly remuneration. While CHWs understood themselves to be volunteers and valued non-material compensation for their work, such as skills-building and feeling part of an international research team, disappointment with the low-salary prevailed, highlighting the complexities of task-shifting in general. CHWs rightly identified that they played a central role in delivering social support, yet did not receive payments aligned to salaries of formal health system employees. The ethical problems of relying on a poor and predominantly female volunteer workforce have been raised repeatedly (Cataldo *et al.* 2015; Topp *et al.* 2015), but since one of the benefits of CHW programmes is their cost-saving potential, no feasible solutions have yet been found.

The limited life-span of the intervention worried CHWs, who felt they were neglecting families with whom they had established relationships of dependence. This reflects findings of other studies. For example, Akintola and Chikoko (2016) report how CHWs felt personally responsible for households’ behaviour in South Africa, and lost motivation when they did not see their efforts resulting in adequate change. Similarly, Loeliger *et al.* (2016) found CHWs used their own resources to assist families with food and transport costs, as they felt personally responsible for the broader well-being of the households they visited (Loeliger *et al.* 2016). In this study, CHWs worried about working within an 18-month time limit per family and felt they were ‘abandoning’ some families still in need of support. Careful preparation for CHWs to ensure they understand their role and what realistic benefits they can bring, as well as help in moderating emotional involvement with community members could help mitigate their sense of failure and prevent burnout.

Finally, the Hermann *et al.* framework includes ‘political will’ for programme scale-up, but this was not an integral component of our intervention. Following the ZENITH trial, a dissemination meeting was held to share the successful results with stakeholders including the National AIDS Council, Ministry of Health, donors and service delivery partners in Zimbabwe. There is no guarantee, however, that the home visit model will be taken up. A recent review of community worker programmes in southern and eastern Africa concluded that CHW programmes are under threat as donor support reduces without compensatory uptake by national health systems (Bemelmans *et al.* 2016). Adaptations will undoubtedly be required for this intervention to operate at larger scale. As is often the case, building political support or refining the design for such scale-up or replication in other settings was beyond the scope of the ZENITH trial’s funding.

## Conclusion

The role of CHWs in delivering community-based HIV care and support is destined to grow, particularly given ambitious targets such as WHO’s 90–90–90 initiative to push near-universal coverage of diagnosis, treatment and adherence among the global population of people living with HIV. Yet numerous challenges remain in managing, retaining and, crucially, providing job satisfaction to the large number of frontline staff that these endeavours will require. This study contributes to the evidence base on CHW-led programmes by describing how one programme interpreted and implemented best-practice criteria.
